# Family Carers of People with Dementia in Japan, Spain, and the UK: A Cross-Cultural Comparison of the Relationships between Experiential Avoidance, Cognitive Fusion, and Carer Depression

**DOI:** 10.1177/08919887221130269

**Published:** 2022-10-19

**Authors:** Naoko Kishita, Hiroshi Morimoto, María Márquez-González, Samara Barrera-Caballero, Carlos Vara-García, Elien Van Hout, Milena Contreras, Andrés Losada-Baltar

**Affiliations:** 1 School of Health Sciences, 83726 University of East Anglia, Norwich, UK; 2 Faculty of Psychology, 12940 Meiji Gakuin University, Tokyo, Japan; 3 Department of Biological and Clinical Psychology, 16722 Universidad Autónoma de Madrid, Madrid, Spain; 4 Department of Psychology, 16776 Universidad Rey Juan Carlos, Madrid, Spain

**Keywords:** caregivers, alzheimer's disease, depression, cultural-comparison, acceptance and commitment therapy

## Abstract

**Objective and research design** This study investigated whether the relationship between experiential avoidance and carer depression is mediated by cognitive fusion using path analysis and whether this model differs between family carers from Japan, Spain, and the UK using multi-group path analysis.

**Results** The whole sample model (*N* = 745) showed a good fit to the data. The direct effect of experiential avoidance on carer depression (*β* = .10) and its indirect effect on carer depression through cognitive fusion (*β* = .15) were significant. Examined variables accounted for 45% of the variance of depression. Multi-group path analysis confirmed the same pattern of indirect path across 3 countries, while the direct path was no longer significant in Spanish and UK samples.

**Conclusion** These findings suggest that targeting cognitive fusion may be particularly critical in culturally diverse carers and pre-emptive efforts to reduce experiential avoidance using psychological techniques may be beneficial among family carers prone to cognitive fusion regardless of cultural differences.

## Introduction

Currently, there are over 55 million people worldwide living with dementia, and the global annual cost of dementia is estimated to be US$ 1.3 trillion.^1^ Informal care provided by family members is estimated to account for half of such annual cost,^1^ suggesting that family carers are an essential taskforce in caring for people with dementia. Although there are various positive aspects of caregiving,^2^ the psychological and physical demands of caregiving can have a significant impact on mental health among family carers. A recent systematic review reported that the pooled prevalence of depression in family carers of people with dementia is 31.2%,^3^ which is substantially higher than the reported prevalence rate in the general population.^4^

Many previous studies have investigated factors associated with depression in family carers of people with dementia. Patient-related factors such as neuropsychiatric symptoms of dementia are known to be strongly associated with carer depression.^5^ In addition, how family carers respond to such caregiving-related stressors through coping strategies are considered to buffer the negative impacts of stressors on carer depression.^6^ However, empirically based models such as the sociocultural stress and coping model^6^ suggest that cultural factors also play a significant role in explaining carer distress, potentially influencing the choice and use of different coping strategies. Therefore, it is important to identify transcultural variables that contribute to carer distress to inform practice, including the development of interventions for culturally diverse carers.

Recent research has highlighted the importance of a currently under-researched psychological dimension that may have a significant impact on carer depression: psychological inflexibility. Psychological inflexibility is considered to emerge from 6 core processes (experiential avoidance, cognitive fusion, attachment to conceptualised past and feared future, attachment to conceptualised self, lack of chosen values, and an inability to broaden and build habits of values-based action), which are the main target of treatment in mindfulness-based interventions such as Acceptance and Commitment Therapy (ACT).^7^ Although all 6 of the processes of psychological inflexibility are interrelated, experiential avoidance (an attempt or desire to control or suppress unwanted internal private events, such as thoughts and feelings, even when doing so is costly or ineffective) and cognitive fusion (the tendency for one’s behaviour to be overly regulated and influenced by cognition) are considered to be more fundamentally linked to each other than the others.^7^ Moreover, both experiential avoidance and cognitive fusion are considered to be pathological processes that are shared across different cultures.^8,9^

Experiential avoidance and cognitive fusion were significantly associated with psychological well-being among family carers of people with dementia in recent studies.^10-15^ These studies also demonstrated that experiential avoidance and cognitive fusion are significantly associated with depression and anxiety in family carers of people with dementia even after controlling for patient-related variables such as neuropsychiatric symptoms^11,12,14^ and the level of independence in activities of daily living (ADL) of the person with dementia.^12^

Previous studies conducted in non-carer samples demonstrated that the combined effects of experiential avoidance and cognitive fusion are more predictive of depression, anxiety, and posttraumatic stress and obsessive-compulsive symptoms than experiential avoidance alone.^16,17^ A recent longitudinal study conducted with a non-clinical sample suggested that the relationship between risk factors (e.g. stressful life events) and future depression and anxiety are mediated by the pathway of experiential avoidance to cognitive fusion but not by the reverse pathway (i.e. cognitive fusion to experiential avoidance) or experiential avoidance alone.^18^ These studies concluded that interventions focused on reducing both, experiential avoidance and cognitive fusion, would be helpful for individuals presenting with depression and anxiety and that pre-emptive efforts to reduce experiential avoidance may be beneficial among individuals prone to cognitive fusion.^16-18^

This study aimed to examine these combined effects of experiential avoidance and cognitive fusion on depression in family carers of people with dementia. Drawing upon the aforementioned studies, we hypothesise that experiential avoidance related to caregiving-related thoughts and feelings (i.e. experiential avoidance in caregiving) will be indirectly associated with carer depression through its association with cognitive fusion. That is, when family carers demonstrate excessive attempts to control caregiving-related thoughts and feelings (experiential avoidance), their behaviour is more likely to be overly regulated by cognition (cognitive fusion), which in turn leads to higher carer depression.

This study also aimed to analyse this proposed model cross-culturally, comparing carers of people with dementia from Japan, Spain, and the United Kingdom (UK). Since dementia is a global health priority worldwide, a cross-cultural comparison of the relationships between experiential avoidance in caregiving, cognitive fusion, and carer depression among family carers from 3 culturally distinct countries may provide important implications in terms of assessment and treatment.

## Methods

### Participants

Participants in this study were family carers of people with dementia and related disorders aged 18 or over recruited in Japan, Spain, and the UK.

#### Japanese Sample (N = 355)

Data from a validation study of the Japanese version of the Experiential Avoidance in Caregiving Questionnaire (EACQ) was used for the Japanese sample.^19^ Participants were recruited through a survey company. To be eligible for this validation study, participants had to be registered with the survey company and to live with their family member with dementia, providing regular care at home (more than five days a week).

#### Spanish Sample (N = 322)

Data collected as part of an ongoing longitudinal study was used for the Spanish sample. To be eligible, participants had to identify themselves as primary carers, providing regular care (at least one hour per day for the last three months) to their family member with dementia or related disorders (e.g. mild cognitive impairment).

#### UK Sample (N = 77)

Screening data collected for an interventional study investigating the feasibility and acceptability of an online self-help ACT programme^20^ was used for the UK sample. Participants had to be primary carers, providing regular care to their family member with dementia, and interested in engaging with online self-help ACT to sign up for screening.

### Measures

#### Relationship to the Care Recipient

Relationship to the care recipient was coded as: 0 = spousal carers, 1 = non-spousal carers.

#### Experiential Avoidance in Caregiving Questionnaire (EACQ)

The original version of the EACQ was developed in Spanish^21^ and has been validated in Japanese.^19^ The English translated version of the EACQ presented in the original validation study,^21^ which has been used in previous research,^20,22^ was used for the UK sample. The 15-item EACQ is specifically designed to assess experiential avoidance in the caregiving context and measures active avoidant behaviours (e.g. “I tend to ‘ignore’ the negative thoughts that come to me about my relative”), intolerance of negative thoughts and emotions towards the relative (e.g. “I cannot bear it when I get angry with my relative”), and apprehension concerning negative internal experiences related to caregiving (e.g. “It is normal for a carer to have negative thoughts about the person they are caring for”). Each item is rated on a 5-point scale from not at all (1) to a lot (5). The total score ranges from 15 to 75, with higher scores indicating higher levels of experiential avoidance. The EACQ has good psychometric properties with good internal consistency.^19,21^ The Cronbach’s alphas for the current study were as follows: Japan = .85, Spain = .73, UK = .78.

#### Cognitive Fusion Questionnaire (CFQ)

The original version of the CFQ was developed in English^23^ and has been validated in Japanese^24^ and Spanish.^13^ The 7-item CFQ assesses cognitive fusion, the tendency for behaviour to be overly regulated and influenced by cognition. An example of items includes “I get so caught up in my thoughts that I am unable to do the things that I most want to do”. Unlike the EACQ, the CFQ is a generic measure and does not assess the influence of specific thoughts related to caregiving. Each item is rated on a 7-point scale from never true (1) to always true (7). The total score ranges from 7 to 49, with higher scores indicating higher levels of cognitive fusion. The CFQ has good psychometric properties with good internal consistency.^13,23,24^ The Cronbach’s alphas for the current study were as follows: Japan = .96, Spain = .92, UK = .94.

### Depression

The original English version of the 9-item Patient Health Questionnaire (PHQ-9)^25^ and the validated Japanese version of the PHQ-9^26^ were used to assess depression in Japan and the UK. The Spanish version^27^ of the 20-item Center for Epidemiologic Studies-Depression Scale (CES-D)^28^ was used to assess depression in Spain. An example of items for the PHQ-9 includes “Feeling down, depressed, or hopeless” and each item of the PHQ-9 is rated on a 4-point scale from not at all (0) to nearly every day (3). The sum of scores of individual items can indicate depression severity of mild (5-9), moderate (10-14), moderately severe (15-19), and severe (20–27). An example of items for the CES-D includes “I felt depressed” and each item of the scale is rated on a 4-point scale from not at all/less than one day (0) to 5-7 days/nearly every day (3). A score of 16 or higher has been widely used as the cut-off point for possible clinical depression. Both the PHQ-9 and CES-D have good psychometric properties with good internal consistency.^25,26,28^ The Cronbach’s alphas for the current study were as follows: Japan = .93, Spain = .77, UK = .86.

### Procedures

Ethical approval was obtained from the relevant ethics committee in each country. Each participant provided written informed consent. Participants completed the questionnaires using a paper and pencil method (Spain) or online (Japan, UK) as part of a larger set of assessments. Participants completed the questionnaires voluntarily without any compensation, except for the Japanese sample that received a redeemable token from the survey company for their participation.

### Statistical Analyses

The original dataset included 782 family carers. First, missing data were checked for independent and dependent variables to be included in path analyses. These included 2 demographic variables (carer age, spousal/non-spousal carer) and 3 key study variables (experiential avoidance, cognitive fusion, carer depression). Since carer age and relationship to the care recipient (spousal carers are at higher risk of depression) are known to have an impact on care depression,^5^ the effects of these demographic variables were also considered in the model.

All statistical analyses were performed using IBM SPSS 28 and AMOS 28. No missing data were identified for any of the variables in the Japanese sample. One or more variables were missing for 26 participants in the Spanish sample. Little's MCAR tests were conducted for all variables, which suggested that data were missing at random. Data for the EACQ were missing for 2 participants in the UK sample. One participant did not have the EACQ data due to a technical error and another participant did not wish to complete the measure. AMOS 28 does not allow conducting certain analyses (e.g. calculation of modifications indices) when there are missing values, and thus participants with missing data were removed from the dataset rather than imputing them as they were minimal (3.6%) and data were missing at random. The final sample included 754 family carers of people with dementia and related disorders. Sample size, gender distribution, mean age, and other demographic variables for each country are presented in Table 1, Table 2 and Table 3.

**Table 1.** Characteristics of Family Carers.

	Total	Japan	Spain	UK	*P*-value	Group comparisons^1^
	N = 754	N = 355	N = 322	N = 77		
Carer age	58.82 (12.45)	54.69 (11.16)	62.28 (12.77)	63.47 (10.64)	<.001	Japan < Spain, UK
Carer gender						
Female	416 (55.2%)	137 (38.6%)	223 (69.3%)	56 (72.7%)	<.001	Japan: male+, female-Spain, UK: male-, female+
Male	338 (44.8%)	218 (61.4%)	99 (30.7%)	21 (27.3%)		
Relationship						
Spousal carers	206 (27.3%)	24 (6.8%)	142 (44.1%)	40 (51.9%)	<.001	^2)^ Japan: spouses-, non-spouses+Spain, UK: spouses+, non-spouses-
Adult children	479 (63.5%)	271 (76.3%)	174 (54.0%)	34 (44.2%)		
Adult children-in-law	34 (4.5%)	34 (9.6%)	—			
Other family members	35 (4.6%)	26 (7.3%)	6 (1.9%)	3 (3.9%)		
Hours of caring per week						
1-10 hours	97 (12.9%)	61 (17.2%)	17 (5.3%)	19 (24.7%)	<.001	^3)^ Japan: less than 40 hours+, more than 41 hours-Spain, UK: less than 40 hours-, more than 41 hours+
11-20 hours	84 (11.2%)	63 (17.7%)	13 (4.1%)	8 (10.4%)		
21-40 hours	182 (24.3%)	137 (38.6%)	40 (12.6%)	5 (6.5%)		
41-80 hours	158 (21.1%)	69 (19.4%)	78 (24.5%)	11 (14.3%)		
81 or more hours	229 (30.5%)	25 (7.0%)	170 (53.5%)	34 (44.2%)		
Living with the care recipient	641 (85.0%)	355 (100%)	238 (73.9%)	48 (62.3%)	<.001	^4)^ Countries associated with the living condition
Length of caring in months	55.39 (45.95)	59.31 (49.78)	51.47 (42.36)	53.64 (40.64)	0.081	—

*Note.* (1) + and – symbols indicate categories that demonstrated the standardised adjusted residuals larger than ±1.96 (i.e. the cell's observed frequency being less/greater than the expected frequency). (2) Two categories were created (i.e. spouses and non-spouses) and compared across 3 countries. (3) Two categories were created (caring for less than/more than 40 hours per week) and compared across 3 countries. 4) Fisher’s exact test was used to determine if there was a significant association between countries and the living condition as the Japanese sample did not have any participants not living with the care recipient.

**Table 2.** Means and Standard Deviations Of Key Study Variables.

	Total	Japan	Spain	UK	*P*-value	Group comparisons
	N = 754	N = 355	N = 322	N = 77		
EACQ (scale range: 15-75)	41.87 (9.16)	39.76 (8.34)	44.31 (9.49)	41.42 (8.95)	<.001	Spain > Japan, UK
CFQ (scale range: 7-49)	21.29 (10.68)	19.37 (10.58)	22.70 (10.60)	24.25 (9.96)	<.001	Japan < Spain, UK
PHQ-9 (scale range: 0-27)	—	8.24 (7.19)	—	7.51 (5.76)	.338	—
CES-D (scale range: 0-60)	—		19.05 (8.19)	—		

*Note.* CES-D= Center for Epidemiologic Studies-Depression Scale, CFQ=Cognitive Fusion Questionnaire, EACQ= Experiential Avoidance in Caregiving Questionnaire, PHQ-9=9-item Patient Health Questionnaire.

**Table 3.** Characteristics of Patients.

	Total	Japan	Spain	UK	p-value	Group comparisons^1^
	N = 754	N = 355	N = 322	N = 77		
Patient Age	81.91 (9.26)	84.37 (9.07)	79.87 (8.88)	79.04 (8.93)	<.001	Japan > Spain, UK
Patient gender						
Female	509 (67.5%)	257 (72.4%)	209 (64.9%)	43 (55.8%)	.012	Japan: male-, female+ UK: male+, female-
Male	239 (31.7%)	98 (27.6%)	107 (33.2%)	34 (44.2%)		
Missing data	6 (0.8%)	—	6 (1.9%)	—		
Dementia type						
Alzheimer’s disease	401 (53.2%)	192 (54.1%)	179 (55.6%)	30 (39.0%)	<.001	^2)^ Japan, Spain: AD+, other dementias- UK: AD-, other dementias+
Other types of dementias	216 (28.6%)	77 (21.7%)	92 (28.6%)	47 (61.0%)		
Dementia type not specified	86 (11.4%)	86 (24.2%)	—			
MCI (probable AD)	26 (3.4%)	—	26 (8.1%)	—		
Neurodegenerative conditions^3^	21 (2.8%)	—	21 (6.5%)	—		
Missing data	4 (0.5%)	—	4 (1.2%)	—		

*Note.* AD=Alzheimer’s disease, MCI=mild cognitive impairment, Unknown=missing data. (1) + and – symbols indicate categories that demonstrated the standardised adjusted residuals larger than ±1.96 (i.e. the cell's observed frequency being less/greater than the expected frequency). (2) Only the first 2 categories were compared across 3 countries (i.e. AD and other types of dementias). (3) Neurodegenerative conditions included other relevant disease such as Huntington's disease

Chi-square tests and one-way analysis of variance (ANOVA) followed by Tukey's HSD post hoc tests were conducted to examine differences in demographic and key study variables (experiential avoidance, cognitive fusion, carer depression) among 3 countries. Depression scores for each country were transformed to z-scores (a measure describing how far a particular value lies from the mean of a normal distribution in terms of standard deviations) as they allow to compare groups across variables that have different measurement units.^29^ Bivariate correlations were calculated to determine the interrelations among key study variables.

**Figure 1.**
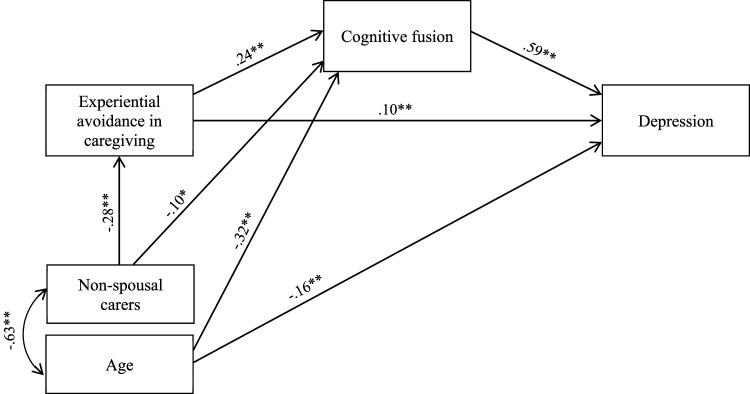
Conceptual overall path analysis model with standardised coefficients. *Note.*^∗∗^*P* < .01 and ^∗^*P* < .05, significant levels of standardised coefficients. The examined variables accounted for 45% of the variance of depression. The errors have been omitted for ease of presentation.

A path analysis was conducted to test the proposed model of an indirect effect of experiential avoidance in caregiving on carer depression through cognitive fusion (see Figure 1). Before the path analysis, tests of skewness and kurtosis were performed for continuous variables (age, experiential avoidance, cognitive fusion, and carer depression). All skewness and kurtosis values were between -1.0 and +1.0, suggesting that the assumption of normality was not violated.^30^ Visual inspection of scatterplots of independent and dependent variables and Cook’s distance were carried out to identify outliers and influential records, and collinearity statistics with the variance inflation factor (VIF) were performed to check multicollinearity. Outlines, influential records, and the issue of multicollinearity were not identified and therefore no further data were excluded. Overall fit of the model was determined using common goodness-of-fit indices including chi-square (*χ*^2^/df < 3, p-value for the model > .05),^31^ comparative fit index (CFI, > .95), the goodness of fit index (GFI, > .95), root mean square error of approximation (RMSEA, < .06), and standardised root mean square residual (SRMR, < .08).^32^ The Hoelter index was also used to test the adequacy of the sample size (Hoelter’s N > 200 suggests the sample size is acceptable).^33^ The indirect effect of experiential avoidance in caregiving on carer depression through cognitive fusion was analysed using 2,000 bootstrap samples and 95% bias-corrected confidence intervals (CI) around the standardised estimate of the effect.

A multi-group path analysis was performed to examine the proposed model across 3 countries. A statistically significant chi-square difference between the unconstrained and structural weights models was checked to determine if the proposed model differed across 3 countries. The path coefficient for the association between experiential avoidance in caregiving and cognitive fusion and between cognitive fusion and carer depression were compared in each pair of countries (Japan vs Spain, Japan vs UK, and Spain vs UK) to examine whether these path coefficients differed by country.

## Results

### Sample Characteristics

Sample characteristics are presented in Table 1, Table 2 and Table 3 with results of chi-square tests and ANOVAs assessing differences among 3 countries. The mean age of participants (N = 754) was 58.82 ± 12.45, with female carers accounting for 55.2% of the sample. More than half of the participants were caregiving adult children (63.5%), caring for a female family member (67.5%) with a clinical diagnosis of Alzheimer’s disease (53.2%).

As shown in Table 1, Japanese carers were significantly younger than carers from Spain and the UK (*F* (2,751) = 41.36, *p* < .001). There were more males and more non-spousal carers than expected in the Japanese sample (gender *χ*^2^ (2, N = 754) = 74.88, *p* < .001; relationship *χ*^2^ (2, N = 754) = 144.75, *p* < .001). As presented in Table 2, Spanish carers reported a significantly higher EACQ score (worse experiential avoidance) than carers from the other 2 countries (*F* (2,751) = 22.06, *p* < .001), while Japanese carers showing a significantly lower CFQ score (less cognitive fusion) than carers from the other 2 countries (*F* (2,751) = 11.80, *p* < .001).

### Correlations of Main Variables

The results of correlation analyses are presented in Table 4. Significant correlations were identified among the EACQ, CFQ, and depression scores in the total sample. The results indicated that higher experiential avoidance in caregiving was associated with higher cognitive fusion and carer depression, and higher cognitive fusion was associated with higher carer depression. The same correlation patterns were observed in Japanese and UK samples. In contrast, the EACQ score was neither significantly correlated with the CFQ nor carer depression in the Spanish sample.

**Table 4.** Correlations between the EACQ, the CFQ and the z-score of depression.

Variables	CFQ				Depression			
	Total	Japan	Spain	UK	Total	Japan	Spain	UK
EACQ	.21**	.28**	.05	.39**	.19**	.32**	.04	.31**
CFQ	—				.65**	.69**	.63**	.63**

*Note.* EACQ=Experiential Avoidance in Caregiving Questionnaire, CFQ=Cognitive Fusion Questionnaire. ^∗∗^*P* < .01 and ^∗^*P* < .05, significance levels of correlations.

### Path Analysis of a Proposed Model

The path analysis among the total sample was conducted as presented in Figure 1. The direct paths from age to experiential avoidance in caregiving and from relationship to the care recipient (non-spousal carer) to carer depression were not statistically significant. Thus, they were omitted to improve the model fit. The final model showed a good fit to the data (*χ*^2^ (2, N = 754) = 4.186, *P* = .123; CFI = .998, GFI = .998; RMSEA = .038; SRMR = .0130; Hoelter’s N = 1078). The examined variables accounted for 45% of the variance of depression.

As shown in Figure 1, the standardised direct effect of experiential avoidance in caregiving on carer depression was .10 (*p* = .001, 95% CI = .05-.15). The standardised indirect effect of experiential avoidance in caregiving on carer depression through its effect on cognitive fusion was .15 (*p* = .001, 95% CI = .11-.19). Higher experiential avoidance in caregiving was associated with higher cognitive fusion, and higher cognitive fusion was associated with higher depression. Increased carer age was associated with lower cognitive fusion and carer depression. Non-spousal carers were more likely to present lower experiential avoidance in caregiving and cognitive fusion as opposed to caregiving spouses.

### Multi-group Path Analysis

**Figure 2.**
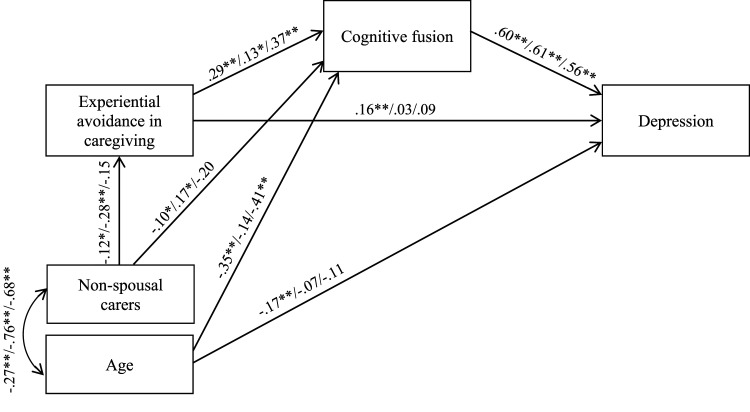
Conceptual path analysis model of cross-cultural differences (Japan/Spain/UK) with standardised coefficients. *Note.*^∗∗^*P* < .01 and ^∗^*P* < .05, significant levels of standardised coefficients. The examined variables accounted for 52%/40%/40% of the variance of depression. The errors have been omitted for ease of presentation.

A multi-group path analysis was conducted to test whether the proposed model differed by country (Figure 2). The fully unconstrained path model provided a good model fit to the data (*χ*^2^ (6, N = 754) = 11.817, *p* = .066; CFI = .994, GFI = .994; RMSEA = .036; SRMR = .0239; Hoelter’s N = 803). The structured weights model demonstrated a less satisfactory fit (*χ*^2^ (20, N = 754) = 47.931, *P* < .001; CFI = .971, GFI = .976; RMSEA = .043; SRMR = .0505; Hoelter’s N = 495). The model comparison suggested that there were significant differences among 3 countries in the whole model (*χ*^2^ (14, N = 754) = 36.114, *P* = .001).

As shown in Figure 2, different patterns of association between demographic variables and key study variables were identified across the 3 countries. The association between age and cognitive fusion was no longer significant in the Spanish sample. The association between age and carer depression was no longer significant in Spanish and UK samples. Relationship to the care recipient (non-spousal carer) and experiential avoidance in caregiving was no longer associated in the UK sample. Non-spousal carer and cognitive fusion were no longer associated in the UK sample. The association between non-spousal carer and cognitive fusion remained significant in the Spanish sample but this relationship became positive rather than negative as it was in the total sample.

The direct path from experiential avoidance in caregiving to carer depression was no longer significant in Spanish and UK samples. The path coefficient for the association between experiential avoidance in caregiving and cognitive fusion and between cognitive fusion and carer depression remained significant across 3 countries. Comparison of single path coefficients demonstrated that the path coefficient for the association between experiential avoidance in caregiving and cognitive fusion was significantly lower in the Spanish sample than in Japanese and UK samples (Spain vs Japan *χ*^2^ (1, N = 677) = 6.317, *P* = .012; Spain vs UK *χ*^2^ (1, N = 399) = 3.863, *P* = .049). No significant difference was observed between Japanese and UK samples (Japan vs UK *χ*^2^ (1, N = 432) = 0.077, *P* = .781). The path coefficient for the association between cognitive fusion and carer depression demonstrated no significant difference among any pairs (Japan vs Spain *χ*^2^ (1, N = 677) = 0.031, *P* =.860; Japan vs UK *χ*^2^ (1, N = 432) =1.472, *P* = .225; Spain vs UK *χ*^2^ (1, N = 677) =1.197, *P* = .274).

## Discussion

A cross-cultural comparison of the relationships between experiential avoidance in caregiving, cognitive fusion, and carer depression was conducted in a sample of 754 family carers of people with dementia and related disorders from 3 culturally distinct countries (Japan, Spain, and the UK). Consistent with a recent study conducted in a non-clinical sample,^18^ the relationship between experiential avoidance in caregiving and carer depression was mediated by cognitive fusion. The tested model explained a substantial variance of carer depression (45%). These findings provide additional support to the importance of experiential avoidance and cognitive fusion as relevant variables for understanding distress among family carers of people with dementia.^10,14,21^

In the multi-group path analysis, group differences in the effects of demographic variables (carer age, relationship to the care recipient) were observed, and some path coefficients between demographic variables and key study variables became no longer significant across 3 countries in different ways. Nevertheless, this was expected since we identified significant differences in demographic characteristics across the 3 samples (e.g. Japanese carers were predominantly young adult children). These differences may have affected the direct path from experiential avoidance in caregiving to carer depression, which was no longer significant in Spanish and UK samples. However, the indirect impact of experiential avoidance in caregiving on carer depression through cognitive fusion remained the same across the 3 participating countries. These findings suggest that experiential avoidance in caregiving and cognitive fusion are inter-related processes underpinning carer depression shared across different cultures, and the combined effects of these variables may explain carer depression better than experiential avoidance alone in culturally diverse carers.

### Clinical and Research Implications

A recent comprehensive meta-analysis showed that psychotherapeutic interventions mainly informed by cognitive behaviour therapy (*g* = .35) and mindfulness-based interventions (*g* =.58) are promising in treating depression among family carers of people with dementia.^34^ However, thus far, many of these published trials have been conducted in Western countries with a predominantly White sample.^35^ The potential transcultural processes underpinning carer depression proposed in this study provide important implications for future interventions targeting culturally diverse carers.

Perhaps the most important clinical implication derived from our findings is that undermining cognitive fusion (the dominance of unhelpful thoughts over behaviour) using psychological techniques, such as defusion (skills to step back from restricting thoughts) may be particularly important in reducing depression among culturally diverse carers. Furthermore, pre-emptive efforts to reduce experiential avoidance using psychological techniques, such as acceptance (skills to allow and embrace difficult thoughts and emotional discomfort) with a specific focus on caregiving-related thoughts and feelings, may be beneficial among family carers prone to cognitive fusion regardless of cultural differences. Defusion and acceptance are the core techniques used in ACT, and studies investigating the effectiveness or feasibility of ACT for family carers of people with dementia are rapidly increasing in recent years, including studies that delivered ACT face-to-face,^36-40^ via telephone,^41^ and online.^20,42,43^

Furthermore, a systematic review of cultural competence in ACT with non-carer samples demonstrated that ACT has been effectively implemented across many countries including low-and middle-income countries.^44^ Future research is recommended to conduct cross-cultural evaluations of ACT with family carers of people with dementia with a specific focus on analysing the role of experiential avoidance and cognitive fusion as mechanisms of change for treatment outcomes.

It is important to note that the score in experiential avoidance in caregiving was significantly higher in Spanish carers compared to carers from the other 2 countries. However, we did not observe significant correlations between the score of experiential avoidance in caregiving and the scores of cognitive fusion and carer depression in the Spanish sample. The path coefficient for the association between experiential avoidance in caregiving and cognitive fusion was significantly lower in the Spanish sample than in Japanese and UK samples. It is possible that experiential avoidance may not play such an important role in explaining depression, particularly when compared with cognitive fusion, among Spanish carers. Alternatively, there may be unexplored factors that have led to differences observed between Spain and the other 2 countries such as sociocultural factors.

Studies conducted in the context of one of the well-established models for explaining carer distress, the sociocultural stress and coping model,^6^ suggests that 2 key factors could potentially affect how carers respond to caregiving-related stressors and distress caused by such stressors: familism and social support. Familism is defined as a strong identification and attachment of individuals with their families and it creates a sense of obligation to take care of one’s family.^45^ Familism is not necessarily always a risk factor and can have positive influences on caregiving experiences, but higher familism has shown to be associated with higher carer depression in Spain.^46^ Further cross-cultural investigations on the role of familism and its association with experiential avoidance may provide a better understanding of differences in processes underpinning carer depression across different countries.

### Methodological Limitations

There are some methodological limitations in this study, which should be considered. We did not consider the impact of caregiving-related stressors such as neuropsychiatric symptoms of dementia and ADL of the person with dementia in the current study. The Spanish sample included a small number of family carers (8.1%) caring for people with mild cognitive impairment who normally tend to present fewer neuropsychiatric symptoms. Future research may benefit from examining the role of caregiving-related stressors in the proposed model.

Although the Hoelter indices suggested that sample size may be acceptable, the sample size of UK carers was small in relation to the other samples. Having an equally large sample for all groups would be ideal for conducting a multi-group path analysis and this may have had an impact on the results. The Spanish sample used a different measure of depression, and the data collection in Spain was conducted face-to-face using a paper and pencil method, while an online approach was used in the other 2 countries. Mental health-related questionnaires are more likely to be influenced by social desirability bias when a face-to-face approach is used than when an online approach is used.^47^ Another notable methodological difference was the use of incentives (redeemable tokens) only in the Japanese sample. These methodological differences may have had an impact on the results. Lastly, this study used a cross-sectional design, which may limit the conclusion regarding the causality between the variables of interest.

## Conclusion

Despite limitations, this study provided a valuable insight into the role of cognitive fusion in the relationship between experiential avoidance in caregiving and carer depression among family carers of people with dementia and related disorders. This indirect impact of experiential avoidance on carer depression through cognitive fusion was observed across 3 countries. Our findings suggest that reducing cognitive fusion is important for treating depression among culturally diverse carers, and pre-emptive efforts to reduce experiential avoidance using psychological techniques, such as acceptance with a specific focus on caregiving-related thoughts and feelings, may be beneficial among family carers prone to cognitive fusion regardless of cultural differences.
